# A case report of atypical anti-glomerular basement membrane disease

**DOI:** 10.1186/s12882-022-03007-y

**Published:** 2022-11-19

**Authors:** Ryo Tamura, Toshiki Doi, Shuma Hirashio, Kensuke Sasaki, Yukinari Masuda, Akira Shimizu, Takao Masaki

**Affiliations:** 1 Department of Nephrology, Hiroshima University Hospital, 1-2-3 Kasumi, Minami-ku, Hiroshima, 734-8551 Japan; 2 Department of Analytic Human Pathology, Nippon Medical School, 1-1-5 Sendagi, Bunkyo-ku, Tokyo, Japan

**Keywords:** Linear deposits of IgG, Atypical anti-GBM disease, Indirect immunofluorescence antibody method

## Abstract

**Background:**

Anti-glomerular basement membrane (anti-GBM) disease is characterized by crescentic necrotizing glomerulonephritis, with linear deposits of immunoglobulin G (IgG) in the GBM. Classic anti-GBM disease is clinically associated with rapidly progressive glomerulonephritis with or without pulmonary hemorrhage. Some patients have a better renal prognosis and milder symptoms than those with classic anti-GBM disease, which is termed atypical anti-GBM disease.

**Case presentation:**

A 43-year-old Japanese woman was admitted to our hospital complaining of hematuria that had persisted for more than one month. Serological examination revealed negativity for anti-nuclear, anti-neutrophilic cytoplasmic, and anti-GBM antibodies. However, renal biopsy showed cellular crescents. Immunofluorescence revealed strong diffuse linear capillary loop staining for IgG. An indirect immunofluorescence antibody method was performed by applying the patient serum to normal kidney tissue to confirm the presence of autoantibodies binding to the GBM. Using this method, anti-GBM antibodies were detected. The patient was treated with high-dose steroids, cyclophosphamide, and plasma exchange. Aggressive treatment resolved proteinuria and hematuria and improved renal function.

**Conclusions:**

Renal biopsy is crucial in the diagnosis of anti-GBM disease, especially when serological tests are negative. Accurately identifying the presence of anti-GBM disease is important to initiate optimal treatment.

## Background

Anti-glomerular basement membrane (anti-GBM) disease presents with necrotizing and crescentic glomerulonephritis caused by autoantibodies against the GBM, which often leads to pulmonary hemorrhage. Anti-GBM glomerulonephritis is a rare disease caused by linear deposition of immunoglobulin G (IgG) autoantibodies against the GBM, most commonly the non-collagenous domain of the alpha-3 chain (α3NC1) of type IV collagen [1, 2]. Classic anti-GBM disease is clinically associated with rapidly progressive glomerulonephritis and pulmonary hemorrhage, a shortened life expectancy, and poor renal prognosis [3–5]. However, some patients have better renal outcomes and milder symptoms than those with classic anti-GBM disease despite strong linear staining for IgG. Moreover, they lack circulating antibodies against α3NC1, which are detected using a commercially available enzyme immunoassay (EIA). The EIA method includes enzyme-linked immunosorbent assays (ELISA), chemiluminescence enzyme immunoassays (CLEIA), and fluorescence enzyme immunoassays (FEIA). This clinical presentation is termed atypical anti-GBM disease [6–10]. Atypical anti-GBM disease is presumably caused by circulating pathogenic immunoglobulin that recognizes different GBM epitopes than those in classic anti-GBM disease.

Classic anti-GBM disease often worsens more rapidly than other types of glomerulonephritis because several glomeruli are damaged simultaneously through the direct mechanism caused by anti-GBM antibodies. Therefore, early diagnosis and aggressive treatment initiation are important. Patients with classic anti-GBM nephritis are usually treated with high-dose steroids, cyclophosphamide, and plasma apheresis [11–13]. The treatment of atypical anti-GBM is controversial, and it is unclear whether the benefits of aggressive treatment outweigh the risks.

Here, we report the case of a patient with acute kidney injury, proteinuria, and hematuria whose renal biopsy showed crescentic glomerulonephritis without circulating α3NC1 antibodies. We diagnosed anti-GBM disease using an indirect immunofluorescence antibody method (IIF). The patient’s proteinuria and hematuria resolved, and her renal function improved with aggressive treatment, including high-dose steroids and plasma exchange.

## Case presentation

A 43-year-old Japanese woman presented to our hospital complaining of hematuria for more than one month. The patient was treated with antibiotics for a suspected urinary tract infection but continued to show hematuria. A cystoscopy was also performed, but there were no abnormal findings. She had no medical history of renal dysfunction and was not taking any regular medication at the time of the first examination. Urinalysis at the first examination showed a urine protein-to-creatine ratio of 1.24 g/gCr, urine red blood cells of > 300/high-power field, and red blood cell casts. Blood sampling revealed progressive renal dysfunction. At admission, the physical examination was unremarkable, but her blood pressure was elevated (146/94 mmHg). Table 1 shows the results of urine and blood data on admission. Urinalysis showed occult blood 3+, urine protein 1+, and urine protein-to-creatinine ratio of 1.42 g/gCr. The number of urine red blood cells was 200–299/high-power field. Her serum blood urea nitrogen was 13.8 mg/dL, serum creatinine was 1.44 mg/dL, and estimated glomerular filtration rate was 33 mL/min/1.73 m^2^. Serological tests revealed no hepatitis virus, normal complement levels (C3: 135 mg/dL, C4: 36 mg/dL), and negative autoantibodies (anti-nuclear, anti-neutrophilic cytoplasmic, and anti-GBM). Anti-GBM antibody testing was performed with the reagent EliA GBM (catalog number: 14–5514-10) and EliA System (Thermo Fisher Scientific, Freiburg, Germany), which uses the human recombinant alpha 3 chain of type IV collagen expressed in insect cells (SF9/baculovirus). This assay was performed on a Phadia 2500EE instrument. On ultrasonography, the kidney size was normal.

**Table 1:** Laboratory results on admission and at the end of treatment

The day of admission	
**Parameter**	**Value**-**(normal range)**
(Urine)	
pH	5
Urine protein/creatinine ratio (g/gCr)	1.42-(< 0.15)
Red blood cell (/HPF	200–299-(< 5)
Oval fat body	–
(Blood)	
White blood cell (/μL)	5780-(3040–8540)
Neutrophil (%)	73.2-(38.3–71.1)
Eosinophil (%)	1.4-(0.2–7.3)
Basophil (%)	0.5-(0.2–2.0)
Lymphocyte (%)	20.4-(21.3–50.3)
Monocyte (%)	4.5-(2.7–7.6)
Red blood cell (104 /μL)	436-(378–499)
Hemoglobin (g/dL)	12.8-(10.8–14.9)
Hematocrit (%)	37.2-(35.6–45.4)
Platelet (104 /μL)	32.1-(15.0–36.0)
AST(U/L)	20-(13–33)
ALT(U/L)	16-(8–42)
Total protein (g/dL)	7.5-(6.7–8.3)
Serum albumin (g/dL)	4.4-(4.0–5.0)
Blood urea nitrogen (mg/dL)	13.8-(8–20)
Creatinine (mg/dL)	1.44-(0.40–0.70)
eGFR (mL/min/1.73 m2)	33-(> 90)
Na (mmol/L)	137-(138–146)
K (mmol/L)	3.9-(3.6–4.9)
Cl (mmol/L)	101-(99–109)
Calcium (mg/dL)	9.4-(8.6–10.4)
Phosphate (mg/dL)	3.3-(2.5–4.7)
Uric acid (mg/dL)	5.1–2.3–7.0)
Plasma glucose (mg/dL)	90-(70–109)
Hemoglobin A1c (NGSP) (%)	5.4-(4.6–6.2)
C-reactive protein (mg/dL)	0.43-(< 0.20)
Immunoglobulin G (mg/dL)	1152-(870–1700)
Immunoglobulin A (mg/dL)	299-(110–410)
Immunoglobulin M (mg/dL)	107-(46–260)
CH50 (IU/mL)	53.2-(30–46)
C3 (mg/dL)	135-(86–160)
C4 (mg/dL)	36-(17–45)
Anti nuclear antigen	Negative
anti-neutrophilic cytoplasmic antibody	Negative
anti-glomerular basement membrane	Negative
HBs-Ag	Negative
HCV-Ab	Negative
The day of the end of treatment	
**Parameter**	**Value**-**(normal range)**
(Urine)	
pH	6.5
Urine protein/creatinine ratio (g/gCr)	<LOD-(< 0.15)
Red blood cell (/HPF)	1–4-(< 5)
Oval fat body	–
(Blood)	
Blood urea nitrogen (mg/dL)	11.4-(8–20)
Creatinine (mg/dL)	0.75-(0.40–0.70)
eGFR (mL/min/1.73 m2)	66-(> 90)

*HPF* high-power field, *AST* aspartate transaminase, *ALT* alanine transaminase, *eGFR* estimated glomerular filtration rate, *IgG* immunoglobulin G, *IgA* immunoglobulin A, *IgM* immunoglobulin M, <LOD below the limit of detection

**Fig. 1:**
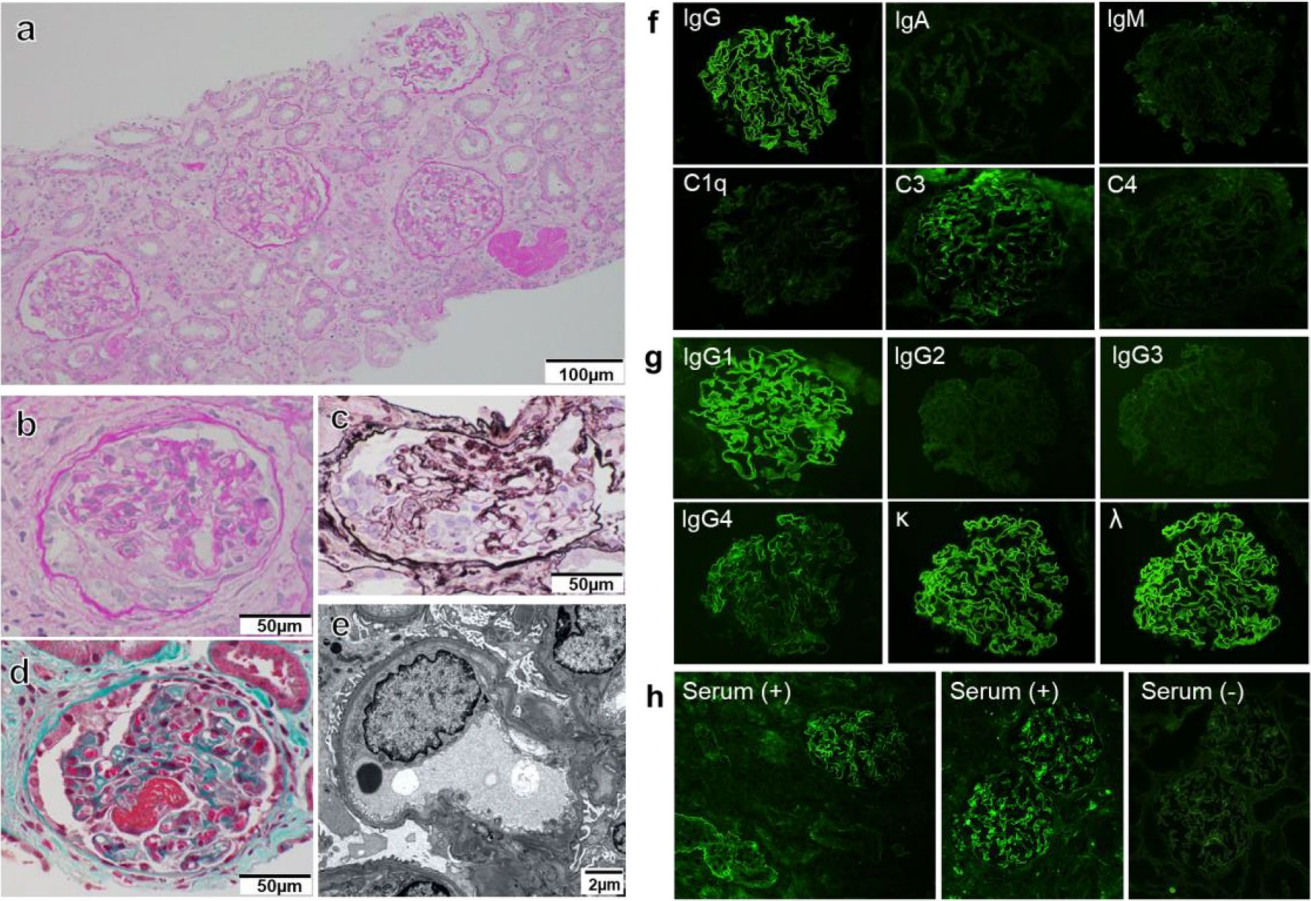
Diagnostic studies. **a** Periodic acid-Schiff (PAS) staining at a low magnification (×200). Crescentic glomerular lesions of **b** PAS, **c** Periodic acid-methenamine-silver, and **d** Masson’s trichrome staining (× 400). Scale bar: 100 μm (a) and 50 μm (b-d). **e** Electron microscopy image. Scale bar: 2 μm. **f** Immunofluorescence studies using frozen sections. Linear capillary loop staining for IgG, and C3 (1+) was positive on glomerular capillaries. **g** IgG1 and IgG4 were positive. Staining for IgG1 (3+) was dominant, and the staining intensity for IgG4 (1+) was rather weak. Staining for IgG2 and IgG3 was negative. Immunofluorescence staining was positive for both kappa and lambda. **h** Immunofluorescence studies using FITC-labeled anti-human IgG (F2020; Dako, Glostrup, Denmark) to determine the presence of IgG binding to the GBM by applying the patient serum to frozen normal kidney tissue after pretreatment in 10 M urea solution for 10 minutes. Anti-GBM antibodies were detected in the Serum (+) specimens in which patient serum was reacted in normal kidney tissue. In contrast, no IgG findings was detected in Serum (−) specimens in which patient serum was excluded in same immunostaining procedures

Using light microscopy, 30 glomeruli were evaluated, none of which showed global sclerosis (Fig. 1a). Cellular crescents were apparent in three glomeruli, but no others showed evidence of fibrous crescents (Fig. 1b–d). In addition to crescentic glomerulonephritis, five glomeruli showed endocapillary hypercellularity. There was no double contour of the capillary walls and hyaline thrombus. Electron microscopy revealed that the foot processes of glomerular epithelial cells were generally preserved, although effacement of the foot process was observed in some basement membranes (Fig. 1e).

Immunofluorescence showed strong, diffuse, linear capillary loop staining for IgG. Immunofluorescence staining for IgG subtypes was performed; IgG1 and IgG4 were positive. Staining for IgG1 (3+) was dominant, and the staining intensity for IgG4 (1+) was rather weak. Staining for IgG2 and IgG3 was negative. C3 (1+) was positive on glomerular capillaries. Immunofluorescence staining was positive for both kappa and lambda (Fig. 1f and g).

Because anti-GBM antibodies were not detected by commercial FEIA, we performed IIF with FITC-labeled anti-human IgG to determine the presence of IgG binding to the GBM by applying the patient serum to normal kidney tissue (Fig. 1h). As a result, anti-GBM antibodies were detected.

**Fig. 2:**
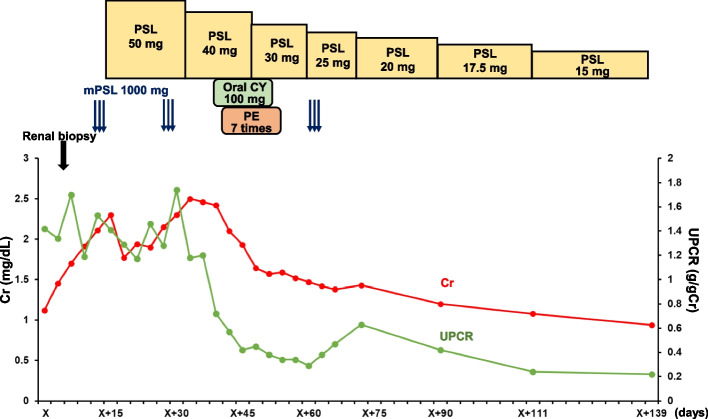
Patient’s clinical course. Changes in serum creatinine (Cr, red lines) and urine protein-to-creatinine ratio (UPCR, green lines). mPSL: methylprednisolone, PSL: prednisolone, CY: cyclophosphamide, PE: plasma exchange

The patient was treated with high-dose steroids, cyclophosphamide, and plasma exchange. Cyclophosphamide was discontinued because of anemia. Aggressive treatment, including three steroid pulses and plasma exchange, decreased proteinuria and hematuria and improved kidney function (Fig. 2). At the end of treatment, proteinuria and hematuria had resolved, and renal function had further improved (Table 1).

## Discussion and conclusions

Our report describes the case of a patient with atypical anti-GBM disease with proteinuria, hematuria, and acute kidney injury. Pathologically, crescentic glomerulonephritis was detected in kidney tissues. Because anti-GBM antibodies binding to alpha3 (IV) NC1 were not detected by FEIA, IIF was used to identify anti-GBM antibodies. Finally, combination therapy with high-dose steroids and plasma exchange was effective for atypical anti-GBM disease with acute kidney injury, resulting in improvements in proteinuria, hematuria, and renal function.

Anti-GBM disease is a type of nephritis that histologically presents as crescentic necrotizing glomerulonephritis, with linear deposits of IgG against the GBM and serological positivity for anti-GBM antibodies. It was first reported by Lerner et al in 1967 [14]. Classic anti-GBM disease is associated with rapidly progressive glomerulonephritis and often pulmonary hemorrhage, shortened life expectancy, and poor renal prognosis [3–5]. Atypical anti-GBM disease presents with better renal outcomes and milder symptoms compared with classic anti-GBM disease, and circulating antibodies to α3NC1 are not detected by EIA in atypical anti-GBM disease.

Anti-GBM antibodies recognize amino acid residues at the N-terminal 17–31 (E_A_) and C-terminal 127–141 (E_B_) of the NC1 domain of the type IV collagen α3 chain as antigen epitopes [15, 16]. In addition to these epitopes, the NC1 domain E_A_ region of the α5 chain has also been reported as an antigenic epitope. These antigen epitopes reside in a hexamer composed of type IV collagen α345 chains and are localized within the basement membrane under normal conditions. Infectious diseases (e.g., influenza), toxic substance inhalation (organic solvents and carbon tetrachloride), and smoking damage the basement membrane of the lungs and kidneys, which exposes antigen epitopes on the α3 and α5 chains and produces anti-GBM antibodies that react to them [17].

The pathological feature of anti-GBM is linear staining of IgG deposited in the GBM, usually accompanied by autoantibodies against structural collagen IV elements of the GBM. Most of these antibodies are directed against α3NC1 and react with epitopes (17–31 = E_A_, 127–141 = E_B_) normally masked by intrachain methionine cross-links [18, 19]. However, antibodies that react with other collagen IV epitopes (e.g., α5NC1 and α4NC1) have also been identified. Furthermore, antibodies that specifically target the α345NC1 hexamer but not the α3NC1 monomer have been reported to be associated with mild nonprogressive glomerulonephritis [20].

Anti-GBM antibodies are detected by IIF, Western blot, and the EIA method, which includes ELISA, CLEIA, and FEIA. The EIA results can be obtained quickly using a fully automated assay. The EIA method has high sensitivity and specificity [21–23]. However, there are cases in which anti-GBM antibodies cannot be detected by EIA; therefore, it is crucial to use IIF or other methods to recognize the presence of anti-GBM disease.

There are several potential reasons why antibodies are not detected by the commercial EIA method. First, the titers of anti-GBM antibodies may be too low. In some reports, low titers of circulating anti-GBM antibodies have been associated with mild proteinuria, hematuria, and renal dysfunction. Low-affinity antibodies to the α3NC1 peptide have also been correlated with reduced crescentic glomerulonephritis and better prognosis. Antibodies with a low affinity can only be detected by sensitive assays, such as Western blot or biosensor experiments, rather than by routine methods [21]. Second, the autoantibodies in some patients may be directed against antigens located on GBM components other than α3NC1. Therefore, the antibodies cannot be detected using routine assays. In the present case, serum anti-GBM antibodies were detected by IIF using normal kidney tissue. Third, similar to other autoimmune diseases, antibody production ceases during the reestablishment of immune homeostasis, and circulating antibodies are destroyed by the liver to a greater extent than tissue antibodies [19]. In these settings, minimal or no antibodies would remain in the circulation, and antibodies would only be present in the GBM.

Classic anti-GBM patients, especially those with initial serum creatinine levels below 5 mg/dL and/or pulmonary hemorrhage, are usually treated with high-dose steroids, cyclophosphamide, and plasmapheresis [13]. However, it remains unclear whether the benefits of these aggressive regimens outweigh the risks in patients with atypical anti-GBM who are clinically and pathologically mild and often without pulmonary hemorrhage. The patient in this case had no pulmonary hemorrhage, but her renal function was declining; therefore, aggressive treatment was administered. Aggressive treatment, including high-dose steroids and plasma exchange, resolved proteinuria and hematuria and improved renal function. Further studies are needed to determine the optimal treatment.

In conclusion, we reported the case of a patient with atypical anti-GBM. If anti-GBM antibodies cannot be detected by EIA, such as ELISA, CLEIA, or FEIA, determining the presence of anti-GBM disease using IIF or other methods to initiate optimal treatment in a timely manner may be useful.

## Data Availability

The datasets used during this case report are available from the corresponding author on reasonable request.

## Abbreviations

IgG: Immunoglobulin G
GBM: Glomerular Basement Membrane
EIA: Enzyme Immunoassay
ELISA: Enzyme-Linked Immunosorbent Assay
CLEIA: Chemiluminescence Enzyme Immunoassays
FEIA: Fluorescence Enzyme Immunoassays
IIF: Indirect Immunofluorescence Antibody Method
Cr: Creatinine
